# RETRACTION: Long Noncoding RNA ZFAS1 Promotes Tumorigenesis and Metastasis in Nasopharyngeal Carcinoma by Sponging Mir‐892b to Up‐Regulate LPAR1 Expression

**DOI:** 10.1111/jcmm.70584

**Published:** 2025-05-09

## Abstract

**RETRACTION**: J. Peng, F. Liu, H. Zheng, Q. Wu, S. Liu, “Long Noncoding RNA ZFAS1 Promotes Tumorigenesis and Metastasis in Nasopharyngeal Carcinoma by Sponging Mir‐892b To Up‐Regulate LPAR1 Expression,” Journal of Cellular and Molecular Medicine 24, no. 2 (2020): 1437–1450, https://doi.org/10.1111/jcmm.14823

The above article, published online on 18 December 2019 in Wiley Online Library (wileyonlinelibrary.com), has been retracted by agreement between the journal Editor‐in‐Chief, Stefan N. Constantinescu; The Foundation for Cellular and Molecular Medicine; and John Wiley & Sons Ltd. The retraction has been agreed after S. Liu raised concerns about the reliability of the results published. Multiple image panels, including Figure 2D, 2E, 5C, 5D, and 7D, were found to be published elsewhere in a different scientific context either before or after the publication of this manuscript. As a result, the conclusions of this manuscript are considered substantially compromised. The authors have been informed about the retraction.

**RETRACTION**: J. Peng, F. Liu, H. Zheng, Q. Wu, S. Liu, “Long Noncoding RNA ZFAS1 Promotes Tumorigenesis and Metastasis in Nasopharyngeal Carcinoma by Sponging Mir‐892b To Up‐Regulate LPAR1 Expression,” Journal of Cellular and Molecular Medicine 24, no. 2 (2020): 1437–1450, https://doi.org/10.1111/jcmm.14823

The above article, published online on 18 December 2019 in Wiley Online Library (wileyonlinelibrary.com), has been retracted by agreement between the journal Editor‐in‐Chief, Stefan N. Constantinescu; The Foundation for Cellular and Molecular Medicine; and John Wiley & Sons Ltd. The retraction has been agreed after S. Liu raised concerns about the reliability of the results published. Multiple image panels, including Figure 2D, 2E, 5C, 5D, and 7D, were found to be published elsewhere in a different scientific context either before or after the publication of this manuscript. As a result, the conclusions of this manuscript are considered substantially compromised. The authors have been informed about the retraction.

